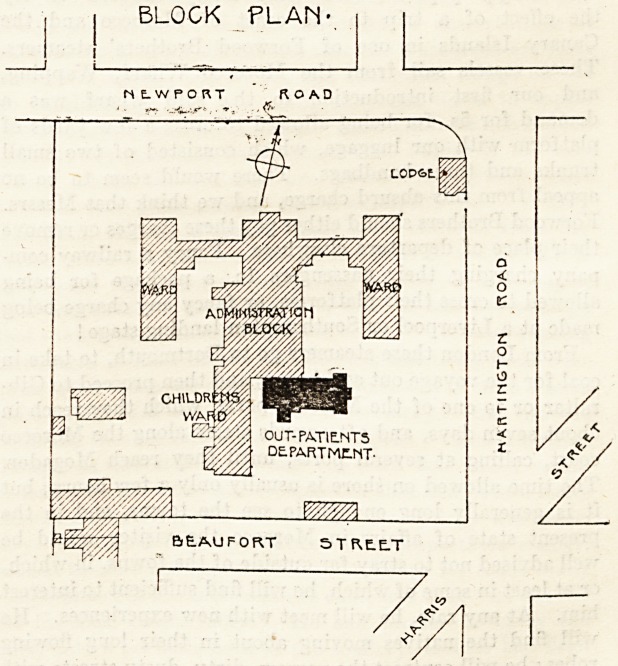# New Out-Patients' Department at the Middlesbrough Infirmary

**Published:** 1906-08-25

**Authors:** 


### NEW OUT-PATIENTS' DEPARTMENT AT THE MIDDLESBROUGH INFIRMARY.

The old Out-patients' Department was erected about thirty years since, and was for some time back found to be quite inadequate for the needs of a hospital which treats something like 6,500 out-patients every year, and which

GROUND PLAN.

number will certainly be exceeded in the near future unless Middlesbrough is unlike most other Infirmaries.

The new department was opened in December last by Sir Samuel Sadler, and after the formal ceremony had been performed, it fell to Lady Sadler to unveil a memorial tablet to the late Angus Macpherson, who had been for thirty-three years Secretary to the institution. The managers deserve a word of praise for thus recording their appreciation of the work done by an old officer—a record all too rare in such institutions.

The site of the Middlesbrough Infirmary, like that of so many town infirmaries, is somewhat restricted; and in planning the Out-patients' Department it was an essential point to interfere as little as possible with the free circulation of air around the wards. It was therefore decided to place the building at the south-east corner of the Administrative Block. It is approached by the porter's lodge entrance in Hartington Road. The waiting-hall is about 33 feet long and 20 feet wide, and is entered by a porch facing the Hartington Road boundary. Next the waiting-hall is the dressing-room, which is very well fitted up with glazed stoneware sinks of various kinds, and beyond it is the consulting-room which can be approached either through a side room (used for special cases) or through the passage opposite the dispensary. After being attended to, patients leave the dressing-room without having to re-enter the waiting-hall. The walls are lined with glazed bricks, all corners are rounded off, and the floors are laid down with maple blocks. The heating is done by low-pressure hot-water coils. The first floor has been utilised for increasing the bedroom accommodation for the nursing staff; and it may be said that the whole department is satisfactorily planned, although it does not seem quite clear to us from which of the dispensary doors, or serving-hatches, the patients will receive their medicines.

The cost of the new work was £1,250, and of this sum £1,222 has been raised. At the opening ceremony Sir Samuel Sadler gave a donation of £10, which reduced the debt on this part of the building to £18. It is not stated in the report before us whether further contributions were received, but it is unlikely that the "large company" separated without subscribing the remaining £18.

The architects were Messrs. Lofthouse and Son, of Middlesbrough.

## Figures and Tables

**Figure f1:**
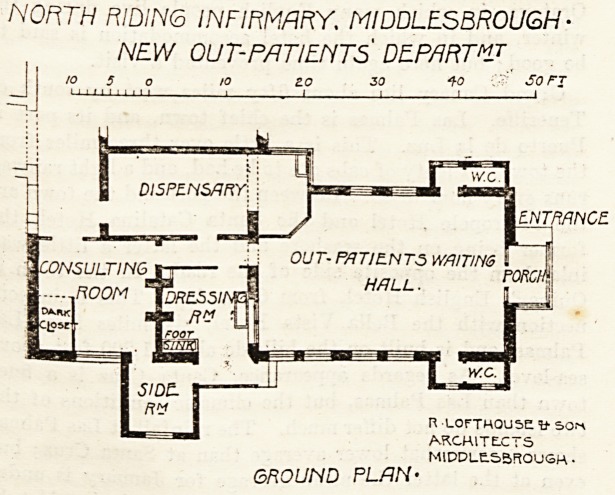


**Figure f2:**